# Toward a Demographic Understanding of Incarceration Disparities: Race, Ethnicity, and Age Structure

**DOI:** 10.1007/s10940-015-9265-6

**Published:** 2015-09-12

**Authors:** Matt Vogel, Lauren C. Porter

**Affiliations:** 1 Department of Criminology and Criminal Justice, University of Missouri-St. Louis, 539 Lucas Hall, St. Louis, MO 63121 USA; 2 OTB – Research for the Built Environment, TU Delft, Delft, The Netherlands; 3 Department of Criminology and Criminal Justice, University of Maryland, College Park, MD USA

**Keywords:** Age structure, Incarceration, Demography

## Abstract

**Objectives:**

Non-Hispanic blacks and Hispanics in the United States are more likely to be incarcerated than non-Hispanic whites. The risk of incarceration also varies with age, and there are striking differences in age distributions across racial/ethnic groups. Guided by these trends, the present study examines the extent to which differences in age structure account for incarceration disparities across racial and ethnic groups.

**Methods:**

We apply two techniques commonly employed in the field of demography, age-standardization and decomposition, to data provided by the Bureau of Justice Statistics and the 2010 decennial census to assess the contribution of age structure to racial and ethnic disparities in incarceration.

**Findings:**

The non-Hispanic black and Hispanic incarceration rates in 2010 would have been 13–20 % lower if these groups had age structures identical to that of the non-Hispanic white population. Moreover, age structure accounts for 20 % of the Hispanic/white disparity and 8 % of the black/white disparity.

**Conclusion:**

The comparison of crude incarceration rates across racial/ethnic groups may not be ideal because these groups boast strikingly different age structures. Since the risk of imprisonment is tied to age, criminologists should consider adjusting for age structure when comparing rates of incarceration across groups.

## Introduction

Although blacks and Hispanics comprise roughly 30 % of the US population, they make up 56 % of the prison population (Humes et al. 2011).^1^ Black/white incarceration disparities are the most pronounced, with black males being incarcerated at nearly seven times the rate of white males. While white males in their 30 s were more likely to have bachelor’s degrees at the close of the century, black males were more likely to have prison records (Pettit et al. 2009). Hispanic/white disparities are noteworthy as well, with Hispanic males being incarcerated at nearly three times the rate of white males (Guerino et al. 2012).^2^ If trends continue, one out of every three black males born in the beginning of the twenty-first century can expect to be behind bars at least once during his lifetime, compared to 1 out of 6 Hispanic males and 1 out of 17 white males (Bonczar 2003).

Disparate rates of imprisonment across racial/ethnic groups are particularly alarming considering the host of negative outcomes associated with going to prison. For instance, incarceration dampens employment prospects (Western 2006), decreases chances of marriage and increases chances of divorce (Western et al. 2001; Huebner 2005), reduces civic engagement (Manza and Uggen 2006), and worsens health (Massoglia 2008a; Hammett et al. 2002; Schnittker and John 2007). Given that blacks and Hispanics are far more likely to experience incarceration than whites, the collateral consequences of imprisonment are also disproportionately afforded to members of these groups. In fact, a handful of studies suggest that incarceration disparities may even contribute to racial inequalities in some of these domains (Massoglia 2008b; Johnson and Raphael 2009).

Although criminologists have been grappling with the underlying causes of incarceration disparities for decades, tests of theoretical explanations are limited (Garland et al. 2008: 26). Prior work in this area is framed within one of two competing schools of thought: (1) higher incarceration rates among blacks and Hispanics reflect true differences in offending (*differential involvement*) or (2) higher incarceration rates reflect discriminatory treatment by the criminal justice system (*differential treatment*) (see Spohn 2000). The general consensus is that both processes are at play. Racial disparities emerge as early as the arrest stage (suggesting differential involvement), but remain pronounced throughout the criminal justice process (suggesting differential treatment after arrest) (Blumstein 1982, 1993; Tonry and Melewski 2009; Baumer 2013).

In this study we investigate a contributing factor that we see as more demographic than theoretical—*differential age structure*. The Hispanic and black populations have a larger portion of individuals at younger, or more “crime-prone,” ages and thus a larger portion of individuals “at risk” of offending. The comparison of non-adjusted incarceration rates—sometimes referred to as *crude rates*—therefore obscures the influence of this compositional difference. For instance, demographers have long-understood the importance of age structure for comparing mortality, morbidity, and fertility risk across groups—especially across countries. Fertility rates are typically higher in younger populations and sickness and death are more common in older populations (see Kitagawa 1955, 1964). Thus, older countries (e.g. Germany) have inflated crude death rates and deflated fertility rates, while younger countries (e.g. Uganda) have inflated fertility rates and deflated mortality rates, making it inappropriate to compare risk using crude rates. A similar logic applies here. In a sense, our argument can be couched in a differential involvement perspective because we argue that Hispanic and black populations may indeed be criminally involved at a higher and more serious level than the non-Hispanic white population. However, our argument departs from this general perspective because we suggest that Hispanics and blacks may be more similarly involved in crime *within* age groups. From this more demographic perspective, it may simply be the case that there are more young blacks and Hispanics than young whites, resulting in higher levels of criminal involvement and by extension larger crude disparities in incarceration.

This study provides a first attempt to discern the contribution of population age structure to racial/ethnic disparities in incarceration. Drawing on data from the Bureau of Justice Statistics and the decennial census, we adjust the black and Hispanic incarceration rates to the white age structure in 2010.^3^ We then employ decomposition techniques to partition these disparities into two components: the percentage attributable to differences in population age structure and the percentage attributable to other sources of variation.

## Race/Ethnicity, Age Structure, and Incarceration

**Fig. 1:**
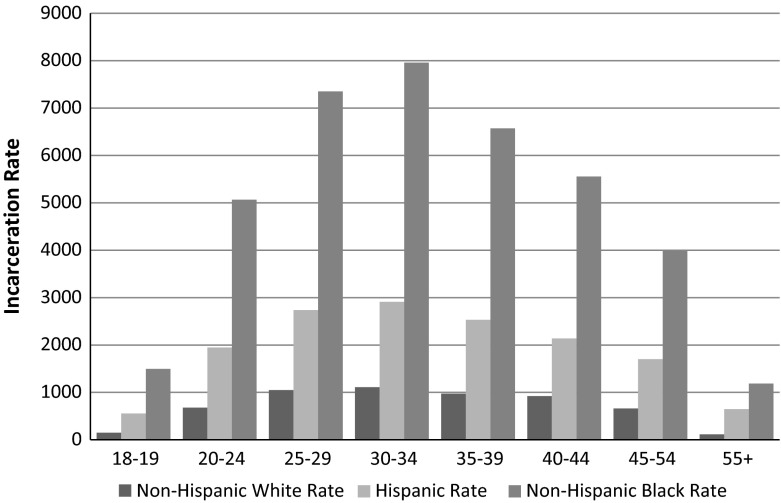
Male incarceration rates per 100,000 by race and age group: 2010

The relationship between age and criminal involvement is one of the most well-established ‘facts’ in criminology. The association is so strong that some have argued it is invariant across cultures and time periods (Hirschi and Gottfredson 1983). The age–crime curve is characterized by a sharp increase in offending through adolescence that peaks in late adolescence and gradually declines thereafter. Although the parameters of the age–crime curve are not identical for all forms of offending, the right-skewed, unimodal shape of the distribution is considered universal (Steffensmeier et al. 1989). This distributional form is apparent when looking at incarceration rates as well (see Fig. 1), although it peaks at later ages since individuals are typically sentenced to prison after committing multiple offenses. In 2010, for example, the age-arrest curve peaked at ages 20–24, while the age-incarceration curve peaked at ages 30–34.^4^

As noted by Cohen and Land (1987), an important implication of the age–crime distribution is that an increase in the proportion of a population in their late teens and early twenties should increase crime rates (p. 173) (see also Fox 2000; Fox and Piquero 2003; Deane 1987). For instance, the precipitous increase in the US crime rate during the 1960s and early 1970s, and the corresponding decreases in the early 1980s have been linked to the aging of the “baby-boom” cohort into, and then out of, crime-prone ages (Cohen and Land 1987; Steffensmeier and Haher 1987, 1991). A similar logic can be applied to understanding differences in criminal involvement and punishment across groups. Demographically speaking, blacks and Hispanics *should* have higher crime rates, and by extension incarceration rates, because young blacks and Hispanics simply comprise a larger portion of those populations.

**Fig. 2:**
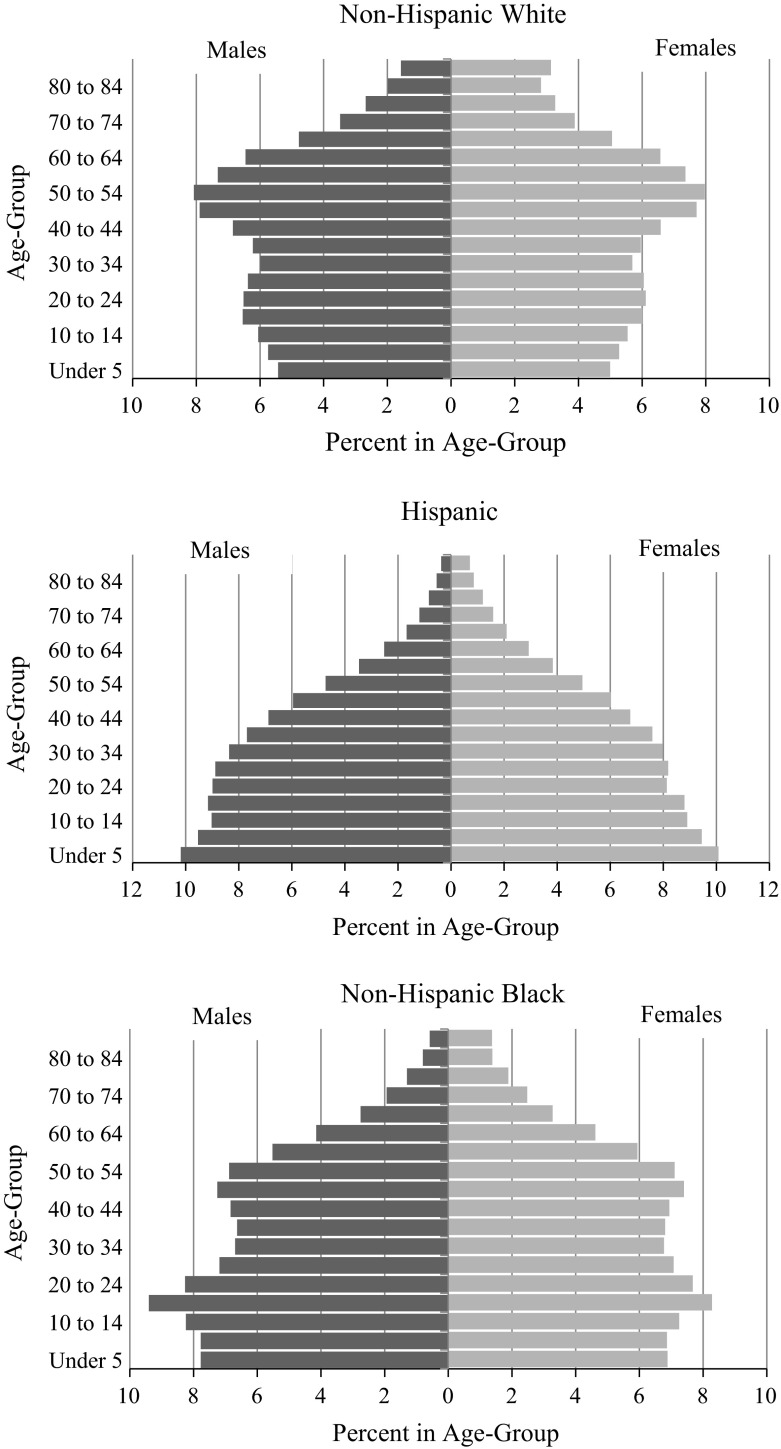
Population age distributions by race/ethnicity: 2010. *Source*: 2010 Decennial Census

To illustrate the differences in age-structure across groups, Fig. 2 presents age pyramids for the Hispanic, white, and black populations in 2010. Each pyramid can be interpreted as two mirrored bar graphs, with one side showing the percent of males in each age group and the other side showing the percent of females in each age group. The younger ages are shown at the bottom, while older ages are at the top. As depicted, the white age distribution resembles a diamond, with the largest proportion of the population clustered between the ages of 40 and 60 (reflecting the baby boomer birth cohort). This pyramid is a typical example of an aging population. The shape is relatively “top heavy” compared to the Hispanic and black populations, which are shaped more like a funnel. The Hispanic and black populations can be characterized as ‘growing’ populations, as their populations are disproportionately clustered at the youngest ages, Hispanics more so than blacks. In an effort to simplistically quantify the differences in age distributions across racial and ethnic groups, we examine dissimilarity indices using the 2010 white population as a base. Similar to the dissimilarity index commonly found in the residential segregation literature, the measure can be interpreted as the proportion of blacks or Hispanics that would need to be redistributed across age categories to generate an age structure similar to the white population in 2010. The index is calculated as:

1 $${\text{D}} = 1/2\sum |{\text{P}}_{{1{\text{j}}}} - {\text{P}}_{\text{ij}} |$$

We use the notation P to refer to population size, the subset i to refer to the racial group in question (such that 1 = white, 2 = Hispanic, and 3 = black) and the subscript j to refer to the age-group (1 = 18–19; 2 = 20–24, etc.). In this case, P_1j_ is the proportion of white males in age group *j* and P_ij_ is the proportion of blacks and Hispanic males in age group *j*, respectively. In 2010, the white–Hispanic index of dissimilarity was 46.9 and the white–black index was 26.7. In other words, nearly half of the Hispanic population would have to be redistributed across age categories in order for the Hispanic and white age structures to look the same, and about one in four black Americans would need to be redistributed for the black population to mirror the age distribution of whites. Further, in 2010 18.6 % of the Hispanic male population and 17.6 % of the black male population were between 15 and 24 years old, the ages traditionally associated with the highest levels of offending. In contrast, only 13.1 % of the white population was concentrated in this age-range.

The stark differences in age structure across racial and ethnic groups indicate that the comparison of crude rates may be problematic. To use an example from the mortality realm, the crude death rate among whites in the US is about 17 % higher than the crude death rate among blacks. At first glance this may seem counterintuitive. However, this difference makes sense given that there are fewer black Americans living to see relatively older ages, where the risk of death is highest. When comparing age-adjusted rates the black mortality rate is actually 20 % higher (Hoyert and Xu 2012). Using another example, blacks in the US are 1.8 % more likely to have diabetes than whites when comparing crude rates. Once again, however, the white population is older on average, meaning there are more people at risk of developing diabetes. After adjusting for age, this difference increases to 3.4 % (CDC 2011).

Drawing on these demographic applications, we examine the contribution of population age-structure to racial and ethnic disparities in incarceration. First, we adjust the Hispanic and black incarceration rates to the white population age structure. This allows us to determine how the Hispanic and black rates would differ if these populations had age structures equal to the white population. Second, we employ a simple decomposition procedure to determine the proportion of the observed disparity in incarceration rates that can be attributed to variation in population age structure across racial and ethnic groups. To our knowledge, this study represents the first investigation into whether differential population age structures contribute to incarceration disparities across racial and ethnic groups in the United States.

## Data and Methods

This study draws on data from the *Prisoners in 2010* report published by the Bureau of Justice Statistics (BJS) and the 2010 decennial census. In the *Prisoners in 2010* report, the BJS provides estimates of the total number of sentenced prisoners on December 31, 2010, by age, sex, race, and Hispanic origin (Guerino et al. 2012, Appendix, Table 13).^5^

In creating the 2010 estimates, separate totals were first generated for federal and state prison populations using the National Prisoner Statistics program (NPS). The NPS, which began in 1925, provides annual administrative counts of inmates serving under state or federal jurisdiction by race, ethnicity and sex. The BJS adjusts these counts according to the annual race-by-sex-by-age distributions reported in the Federal Justice Statistics Program (FJSP) and the National Corrections Reporting Program (NCRP) in 2010. The differences between administratively reported race data and self-reported race data were then adjusted for using the 2008–2009 National Inmate Survey.^6^ Finally, age-specific imprisonment rates for each race-by-sex group were calculated by dividing the estimated number of sentenced prisoners within each age group by the estimated number of US residents in each age group on January 1, 2011. This quotient was multiplied by 100,000 and rounded to the nearest whole number (see Guerino et al. 2012: pp. 8–9 for more information).^7^

In generating the standardized rates and decompositions, we link the BJS data with information provided by the 2010 decennial census data. Specifically, we employ national population counts for males by age and race/ethnicity. These data are based on self-reports and Hispanic ethnicity is treated as distinct from race. Respondents first indicate whether they are of “Hispanic, Latino, or Spanish origin” and then they indicate their race in the next question. As in previous years, the Bureau of the Census reported undercounting the black and Hispanic population by 2.1 and 1.5 %, respectively.^8^

## Analytic Strategy

### Estimating Race-Specific Incarceration Rates

We begin by estimating the crude incarceration rates for the white, black, and Hispanic male population over the age of 18.^9^ These rates reflect the non-adjusted, overall incarceration rates in 2010. Table 1 presents age-specific male incarceration rates for 2010. In this table, P_ij_ refers to the number of males in each race/ethnicity, *i* by age-group *j*, P refers to the race/ethnicity-specific population of males over the age of 18, P_ij_/P_i_ refers to the proportion of the race/ethnic-specific male population over the age of 18 in each age group, and E_ij_ is the estimated number of race/ethnic specific males under federal or state jurisdiction in each age group. The age-by-race-specific incarceration rates, denoted as T_ij_, are computed as:

2 $${\text{T}}_{\text{ij}} = \frac{\text{Eij}}{\text{Pij}} \times 100{,}000.$$

These rates can be interpreted as the number of male prisoners in each race/ethnic group per 100,000 males in each race/ethnic group in the general population. For instance, there were 149.3 white males between the ages of 18 and 19 incarcerated per every 100,000 white males in this age group in the general population.

**Table 1:** Male population, estimated prisoners under state and federal jurisdiction, and estimated incarceration rates per 100,000 by age and race/ethnicity in 2010

Age range	Non-Hispanic White				Non-Hispanic Black				Hispanic			
	P_ij_^a^	P_ij_/P_i_	(E_ij_)	(T_ij_)	P_ij_^a^	P_ij_/P_i_	(E_ij_)	(T_ij_)	P_ij_^a^	P_ij_/P_i_	(E_ij_)	(T_ij_)
18–19	2612	0.042	3900	149.3	694	0.055	10,400	1497.4	955	0.055	5300	554.9
20–24	6308	0.101	42,800	678.4	1484	0.117	75,200	5066.5	2302	0.132	44,800	1945.9
25–29	6170	0.099	64,700	1048.5	1290	0.102	94,900	7351.5	2276	0.131	62,300	2737.1
30–34	5799	0.093	64,300	1108.7	1202	0.095	95,700	7959.4	2143	0.123	62,400	2911.7
35–39	6025	0.096	58,800	975.8	1191	0.094	78,300	6573.2	1970	0.113	49,900	2532.0
40–44	6630	0.106	61,200	923.1	1227	0.097	68,200	5554.8	1763	0.101	37,700	2137.6
45–49	7640	0.100	59,400	777.4	1302	0.103	60,000	4607.3	955	0.090	28,600	1875.4
50–54	7817	0.102	43,200	552.6	1236	0.097	41,100	3323.9	2302	0.072	18,000	1486.4
55–59	7089	0.093	24,700	348.4	991	0.078	21,400	2158.4	2276	0.053	9300	1045.9
60–64	6244	0.082	14,700	235.4	744	0.059	9300	1249.4	2143	0.038	4600	712.6
65+	14,031	0.184	13,500	96.2	1324	0.104	5600	422.8	1970	0.070	3600	304.6
P: ∑P_a_	76,371				12,690				16,863			
E: ∑E_a_	451,200				560,100				326,500			
T:	590.8				4413.7				1936.1			

P_a_, males per age group; P, total male population; E_a_, incarcerated males per age group; T_a_, age-specific male incarceration rate

^a^In thousands

Second, we calculated the race-specific incarceration rates, which are estimated as:

3 $${\text{T}}_{\text{i}} = \frac{{\sum {\text{Eij}}}}{\text{Pi}} \times 100{,}000.$$

This reflects the total number of male prisoners in each race/ethnic group per ery 100,000 males in the population. Consistent with prior research, we find that the crude Hispanic incarceration rate is roughly twice the white rate in 2010, while the black incarceration rate is six times higher than the white rate.

### Age-Standardized Incarceration Rates

We next attempt to eliminate the confounding effect of population age structure by adjusting the crude black and Hispanic incarceration rates using the white male population as a standard. These age-adjusted incarceration rates can be interpreted as the hypothetical black and Hispanic incarceration rates that would have occurred if the black and Hispanic populations had an age distribution equal to that of the white population (McGehee 2004).

We adjust the Hispanic and black incarceration rates using a direct standardization technique. In the procedure, the standardized rate, T* is computed as:

4 $${\text{T}}^{*} = \frac{{\sum \left( {{\text{Tij}} \times {\text{P}}1{\text{j}}} \right)}}{{{\text{P}}1}} \times 100{,}000$$

where *T*_*ij*_ is the age-specific incarceration rate for Hispanics or blacks, *P*_*1j*_ represents the white population total in each age group, and P_1_ is the total white population. Essentially, each non-white age-specific incarceration rate is multiplied by the white population count for that age group. The sum of T_ij_ × P_1_ reflects the number of black and Hispanic prisoners we would expect to observe if the black and Hispanic populations had an age distribution identical to the white population. Dividing this sum by the white population generates the adjusted incarceration rate. It is important to note that the adjusted rate has no intrinsic meaning. It is only meaningful when compared with other adjusted rates calculated on the same standard. Therefore, we compute relative differences between the crude and adjusted white, Hispanic, and black incarceration rates.

### Decomposing the Differences in Rates

In the second analysis, we employ a simple technique to decompose the *absolute* difference in incarceration rates into two components: the percentage of the difference that can be attributed to population age structure and that due to differences in age-specific rates of incarceration (Kitagawa 1955, 1964). To begin, the difference between the Hispanic and white incarceration rates can be expressed as the sum of two major components:

5 $${\text{T}}_{2} -{\text{T}}_{1} = \sum \frac{{\frac{{{\text{P}}2{\text{j}}}}{{{\text{p}}2}} + \frac{{{\text{P}}1{\text{j}}}}{{{\text{P}}1}}}}{2}\left( {{\text{T}}2{\text{j}} - {\text{T}}1{\text{j}}} \right) + \sum \frac{{{\text{T}}2{\text{j}} + {\text{T}}1{\text{j}}}}{2} \left( { \frac{{{\text{P}}2{\text{j}}}}{{{\text{P}}2}} - \frac{{{\text{P}}1{\text{j}}}}{{{\text{P}}1}}} \right)$$

In this equation, *P*_1_ and *P*_2_ refer to the total population for whites and Hispanics respectively. T_1j_ and T_2j_ refer to the age-specific incarceration rates for whites and Hispanics and P_1j_ and P_2j_ refer to the population of whites and Hispanics in each age group. T_2_ − T_1_ refers to the absolute difference in crude incarceration rates between the white and the Hispanic population. The first component is the proportion of the disparity due to differences in age-specific rates. It is calculated as the sum of the difference in rates between groups (in this case, Hispanics and whites) at each age group weighted by the average proportion at that age across groups. This can be interpreted as the absolute difference in crude incarceration rates attributable to differences in the actual rates (in other words, the portion of the variation that *cannot* be explained by differences in age structure). The second component can be interpreted as the proportion of the difference attributable to differences in age structure. It is calculated as the difference in proportion of Hispanics and whites in each age group weighted by the average incarceration rate across groups at each age. These separate components are divided by the T_2_ − T_1_ to ascertain the portion the disparity that is due to differences in rates and differences in age structure (see Eqs. 6 and 7).^10^

6 $$\left( { \sum \frac{{\frac{{{\text{P}}2{\text{j}}}}{{{\text{P}}2}} + \frac{{{\text{P}}1{\text{j}}}}{{{\text{P}}1}}}}{2}\left( {{\text{T}}2{\text{j}} - {\text{T}}1{\text{j}}} \right)} \right)/\left( {{\text{T}}_{2} - {\text{T}}_{1} } \right) = {\text{Proportion}}\,{\text{due}}\,{\text{to}}\,{\text{difference}}\,{\text{in}}\,{\text{rates}}$$

and

7 $$\left( {\sum \frac{{{\text{T}}2{\text{j}} + {\text{T}}1{\text{j}}}}{2} \left( { \frac{{{\text{P}}2{\text{j}}}}{{{\text{P}}2}} - \frac{{{\text{P}}1{\text{j}}}}{{{\text{P}}1}}} \right)} \right)/\left( {{\text{T}}_{2} - {\text{T}}_{1} } \right) = {\text{Proportion}}\,{\text{due}}\,{\text{to}}\,{\text{differences}}\,{\text{age}}\,{\text{structure}} .$$

## Results

Table 2 presents the results of the age-standardization and decomposition procedures. The second row in this table presents the crude (non-adjusted) incarceration rates for each race/ethnic group (∑E_ij_/P_i_). The third and fourth rows present the total difference in incarceration rates between whites and the other race/ethnic groups (T_i_ − T_1_) and the percent difference in the crude incarceration rates between whites and the other groups respectively ((T_1_ − T_i_)/T_1_). These figures indicate non-Hispanic blacks were incarcerated at 6.47 times the rate of whites and Hispanics were incarcerated at 2.27 times the rate of whites.

**Table 2:** Components of age standardization and decomposition procedures

	NH–White	NH–Black	Hispanic
*Age*-*standardization*			
Total population: P^a^	76,371	12,690	16,863
Crude incarceration rate (CIR): ∑E_ij_/P_i_	590.8	4413.65	1936.13
Difference in CIR: T_i_ − T_1_	–	3823.85	1345.33
% Difference in CIR: (T_i_ − T_1_)/T_1_	–	6.47	2.27
∑t_ij_P_1j_	–	2,940,562	1,190,196
Age adjusted incarceration rate: ∑T_ij_P_1j_/P_1_	590.8	3850.36	1558.44
Difference in adjusted rate	–	3260.26	967.64
% Difference in adjusted rate	–	5.51	1.63
*Decomposition analyses*			
Difference due to rate: $\sum \frac{{{\text{Tij}} + {\text{T}}1{\text{j}}}}{2} \left( { \frac{\text{Pij}}{\text{Pi}} - \frac{{{\text{P}}1{\text{j}}}}{{{\text{P}}1}}} \right)$	–	3504.19	1082.79
Difference due to age: $\sum \frac{{\frac{\text{Pij}}{\text{Pi}} + \frac{{{\text{P}}1{\text{j}}}}{{{\text{P}}1}}}}{2}\left( {{\text{Tij}} - {\text{T}}1{\text{j}}} \right)$	–	318.66	262.54
Proportion of difference in CIR due to rate^b^	–	0.916	0.805
Proportion on difference in CIR due age^b^	–	0.083	0.195

^a^In thousands

^b^Relative to the non-Hispanic white male population

The age-standardized rates (∑T_ij_P_1j_/P_1_) demonstrate that a non-trivial portion of these disparities can be attributed to differences in population age structure. For instance, the incarceration rate for a population experiencing the same age-specific incarceration rates as the Hispanic population with an age distribution equal to the white population would be 19.5 % lower than the crude Hispanic incarceration rate [(1936.13 − 1558.44)/1936.13]. Similarly, the incarceration rate for a population experiencing the same age-specific incarceration rates as the black population with an age distribution equal to the white population would be 12.8 % lower than the black crude incarceration rate in 2010. In other words, these results suggest that the Hispanic incarceration rate would be 19.5 % lower if the Hispanic population had an age structure identical to that of the white population, and that the black incarceration rate would be 12.8 % lower. Notably the differences between the crude and adjusted rates are larger for Hispanics than blacks, suggesting that population age structure may have a greater influence on the incarceration disparity between Hispanics and whites.^11^

These findings suggest that the size of the disparity in incarceration rates between Hispanic males and white males would have been roughly 28 % lower [(2.27 − 1.63)/2.27] if the Hispanic population had an age structure equal to white population. Likewise, the disparity between blacks and whites would have been roughly 14.4 % lower in 2010 if the black population had an age structure equal to white population. Thus, holding constant the prevailing age-specific incarceration rates, a portion of racial and ethnic disparities in incarceration can be attributed to differences in population age structure across groups. The results of the decomposition analysis comport with the age-standardization procedures and once again underscore the importance of population age structure in the observed racial and ethnic disparities in incarceration. These analyses also demonstrate that age differences play a much larger role in the Hispanic/white disparity than the black/white disparity. 19.5 % of the disparity between Hispanic and white males can be attributed to age structure differences. Conversely, roughly 8.3 % of the difference in crude incarceration rates between blacks and whites can be attributed to age structure.

## Conclusion

This study takes a first step toward understanding the contribution of population age structure to the observed racial and ethnic disparities in incarceration. Demographers are mindful that differential age structures obscure comparisons across groups with respect to morbidity, mortality, and fertility patterns. Similarly, we argue that the comparison of crude rates of incarceration across racial and ethnic groups may be problematic, given that the risk for incarceration is intricately linked to age, and the age distribution of the US population varies substantially across these populations. Results of the standardization and decomposition procedures demonstrate that the relatively younger ages of the black and Hispanic populations contribute to racial and ethnic disparities in incarceration. Specifically, age-standardization procedures suggest that the black/white disparity would be about 14 % lower and the Hispanic/white disparity about 28 % lower if these populations had age structures comparable to the white population. Further, around 20 % of the Hispanic/white and 8 % of the black-white disparity can be attributed to differences in population age structures.

As a practical and methodological contribution, our results suggest that criminologists should consider incorporating these techniques when assessing racial disparities in punishment and in criminal involvement. Demographers have long-recognized the merit of age-standardization and decomposition techniques for quantifying and understanding risk across populations, but other social sciences have been slower to adopt these techniques when comparing crude rates across groups. Building on this topic alone, there are several fruitful directions researchers may consider to further understand the role of population age-structure in shaping racial and ethnic disparities in incarceration. For one, researchers might apply these techniques to specific offense types to further explore issues of differential treatment versus differential involvement. For instance, research suggests that differential treatment is most pronounced for less serious crimes such as drug offenses (Blumstein 1982, 1993; Austin and Allen 2000), but it remains unclear how differences in age structure may account for disparities in these crime types.

Scholars might also consider the role of age structure in explaining differences in incarceration disparities over time and between states. For one, population dynamics may explain increasing racial and ethnic disparities over time. The white population is aging at a relatively quick rate, while the Hispanic population has remained relatively ‘young’ and by all indications will continue to do so for the foreseeable future. How these trends have and will continue to shape incarceration disparities remains unclear. Second, there is considerable variation in population composition—in terms of both race and age—across the United States. Thus, it also bears to reason that these compositional differences should account for at least part of the racial disparity in state-level incarceration rates.

To this end, we carried out two supplemental analyses to provide a better idea of how age structure may operate across states and over time to shape disparities. Currently 29.4 % of the Hispanic male population is under the age of 15, compared to 24 % of the black male population and 17.3 % of the white male population. In relative terms, almost twice as many young Hispanic males will enter into the high-crime ages over the next decade than young white males. Assuming stable population dynamics and stable race and age-specific incarceration rates, the incarceration *disparity* between Hispanics and whites could increase by roughly 23 % due to shifts in age structure alone. Likewise, the black/white disparity may increase by as much as 15 % (full details of analyses and results available upon request). Using data from the NCRP and the decennial census for 2010, we also find that state rankings for racial/ethnic incarceration disparities do not differ substantially when using standardized versus crude rates, meaning that states with the largest crude incarceration disparities also exhibit the largest age-adjusted disparities. However, there is a relatively major change that we see as pertinent. When using crude rates, 23 % of states included do not have a Hispanic/white disparity. Conversely, this percentage jumps to 43 % when using age-standardized rates, meaning that when standardizing by age 43 % of states had a *white/Hispanic* disparity in 2010 (see “Appendix 2”).

It is important to note that while we find a component of the racial/ethnic disparities in incarceration are attributable to differences in age structure, a larger component is not. Our approach does not enable us to speak to what is driving this unexplained portion; however, it could be indicative of differential involvement (higher rates of offending among blacks and Hispanics, even after adjusting for age) or differential treatment (a greater propensity for Hispanics and blacks to be incarcerated regardless of offending behaviors).

In sum, this analysis provides preliminary evidence that population age structure contributes to incarceration disparities across racial and ethnic groups in the United States. We suspect that age structure is a salient force in shaping disparities at other stages of the criminal justice process as well, such as in rates of criminal offending and victimization. As such, we caution researchers against accepting differences in crude rates at face value and encourage researchers to adjust for age structure where possible.

## Appendix 1

See Tables 3 and 4.

**Table 3:** Components of age standardization with Hispanic population as standard

Age-standardization	Hispanic	NH–White	NH–Black
Total population: P^a^	16,863	76,371	12,690
Crude incarceration rate (CIR): ∑E_ij_/P_i_	1936.13	590.8	4413.65
Difference in CIR: T_i_ − T_2_	–	−1345.3	−2477.71
% Difference in CIR: (T_i_ − T_2_)/T_2_	–	−0.69	−1.27
∑t_ij_P_2j_	–	124,484	839,125
Age adjusted incarceration rate: ∑T_ij_P_2j_/P_2_	1936.13	738.21	4975.9
Difference in adjusted rate	–	−1197.92	−3042.7
% Difference in adjusted rate	–	−0.62	1.57

^a^In thousands

**Table 4:** Components of age standardization with non-Hispanic Black population as standard

Age-standardization	NH–Black	NH–White	Hispanic
Total population: P^a^	12,690	76,371	16,863
Crude incarceration rate (CIR): ∑E_ij_/P_i_	4413.65	590.8	1936.13
Difference in CIR: T_i_ − T_3_	–	−3823.2	2477.7
% Difference in CIR: (T_i_ − T_3_)/T_3_	–	−0.87	−0.56
∑t_ij_P_3j_	–	84,361	221,979
Age adjusted incarceration rate: ∑T_ij_P_3j_/P_3_	4413.65	664.8	1749.3
Difference in adjusted rate	–	−3784.5	2264.4
% Difference in adjusted rate	–	−0.85	−0.60

^a^In thousands

## Appendix 2

See Table 5.

**Table 5:** Incarceration disparities by state

Ranking	B/W crude disparity	B/W adjusted disparity	H/W crude disparity	H/W adjusted disparity
1	Minnesota	Minnesota	Massachusetts	Massachusetts
2	New Jersey	New Jersey	Pennsylvania	Pennsylvania
3	Massachusetts	Massachusetts	North Dakota	North Dakota
4	Montana	New York	New York	New York
5	Pennsylvania	Pennsylvania	Nebraska	Colorado
6	New York	Montana	Colorado	Nebraska
7	Nebraska	Nebraska	New Hampshire	South Dakota
8	North Dakota	Colorado	South Dakota	Minnesota
9	Colorado	North Dakota	Minnesota	Utah
10	South Dakota	Utah	Rhode Island	New Hampshire
11	Kansas	Kansas	New Jersey	Rhode Island
12	Rhode Island	Wyoming	Utah	Montana
13	Utah	California	Montana	New Jersey
14	New Hampshire	South Dakota	Wyoming	Wyoming
15	Wyoming	Rhode Island	Kansas	California
16	California	Ohio	California	Texas
17	Maine	Maryland	Oregon	Kansas
18	Maryland	Oregon	Texas	Oregon
19	Ohio	Washington	Ohio	Idaho
20	Oregon	Michigan	Idaho	Ohio
21	Washington	Maine	Nevada	**Nevada**
22	Michigan	New Hampshire	Washington	**Washington**
23	Indiana	Indiana	Indiana	**Oklahoma**
24	North Carolina	North Carolina	North Carolina	**Indiana**
25	Texas	Texas	Oklahoma	**North Carolina**
26	Delaware	South Carolina	Georgia	**Alaska**
27	Florida	Oklahoma	**Alaska**	**Georgia**
28	South Carolina	Delaware	**Delaware**	**Missouri**
29	Nevada	Nevada	**South Carolina**	**Delaware**
30	Tennessee	Tennessee	**Missouri**	**Florida**
31	Georgia	Florida	**Florida**	**South Carolina**
32	Oklahoma	Georgia	**Tennessee**	**Tennessee**
33	West Virginia	Kentucky	**Mississippi**	**Mississippi**
34	Kentucky	Missouri	**Kentucky**	**Kentucky**
35	Missouri	West Virginia	**West Virginia**	**West Virginia**
36	Alabama	Alabama		
37	Idaho	Idaho		
38	Mississippi	Mississippi		
39	Alaska	Alaska		

Bold states have a higher non-Hispanic white incarceration rate than Hispanic rate

## Footnotes

1 For simplicity’s sake, we use black, white, and Hispanic to refer to these population groups, where black refers to non-Hispanic black and white refers to non-Hispanic white.

2 The risk of incarceration also varies significantly by sex, as 93 % of all prisoners are male (Carson and Sabol 2012). The sex/gender gap in crime has been attributed to biological, psychological, and social differences between men and women (Moffitt 2001). Although there is evidence that the gap may be closing, evidence suggests this is due to decreases in male offending rather than increases in female offending (Lauritsen et al. 2009).Given the stark differences in incarceration across groups, we confine our analysis to males. However, the carried out the analyses using the total population as well and found no differences in our estimates (results available upon request).

3 We focus on disparities across black, white, and Hispanic groups because age-specific rates of incarceration are only provided for these groups in the Prisoners in 2010 report (see Guerino et al. 2012: Appendix Table 13).

4 The inmate population is also aging. According to Porter et al. (2015), the median age of inmates increased from 27 in 1974 to 34 in 2004. The rise in the elderly population has been attributed to the increase in admission rates of older inmates (Luallen and Kling 2014).

5 Estimates are provided for age-*groups* rather than specific ages and therefore our analysis is limited to age-groups as well.

6 The National Inmate Survey has been conducted annually since 2003 as part of the BJS National Prison Rape Statistics Program to gather data on the prevalence of sexual assault in correctional facilities. However, inmates are also asked to report demographic information as well.

7 The methodology used by the NCRP improved substantially in 2010. Given these improvements, however, the BJS cautions researchers from comparing 2010 data with earlier years (see Guerino et al. 2012: p. 8). Therefore, we confine our analysis to 2010. It is also important to note that these data reflect rates of confinement, rather than new admissions. Thus, these data capture the stock of the prison population, rather than the flow. A potential issue here is that the population base used in the denominator of the rates may not accurately reflect the demographic composition of the population at the time an inmate first entered prison. However, the median sentence length is just over 2 years, so it is unlikely that this will significantly bias the results presented here.

8 Pettit (2012) discusses the difficulties in enumerating young black and Hispanic men. With respect to this study in particular, the age structure differences across groups are probably more dramatic than what is shown because young black and Hispanic men are especially likely to be undercounted by the census. However, the limitation could also slightly inflate racial disparities at younger ages, since non-incarcerated minority men may be “missing” from the denominator when constructing incarceration rates. These undercounts may overstate disparities in incarceration rates between whites and blacks in particular. Young black males are disproportionately likely to (1) end up behind bars and (2) be ‘missing’ from the census population-enumeration used in the denominator. This is an unfortunate limitation for any researcher trying to study risk among the black population.

9 The NPS only reports on adult populations, hence our decision to focus only on individuals over the age of 18. As such, our estimates of the total incarceration rate will differ from incarceration rates reported elsewhere that use the *entire* population in the denominator. We also confine our analysis to the male population, given the markedly low counts of female inmates across certain age-by-race groups.

10 An alternative decomposition strategy common in the criminological literature (e.g. Steffensmeier and Harer 1987; Levitt 1999) is to estimate the contribution of population age structure to the *relative* difference in incarceration rates. In this technique, the contribution of population-age structure is estimated as the difference between the crude and adjusted Hispanic incarceration rates or T_2_ − T*_2_. The contribution of population age structure to the relative difference in incarceration rates can then be estimated by dividing this figure by the total difference in crude rates between Hispanics and whites or (T_2_ − T*_2_)/(T_2_ − T_1_). In general, the weighted average approach presented in Eq. 5 will produce more conservative estimates of the contribution of population age structure to the difference in rates. For a detailed discussion of the key differences between these decomposition strategies, we refer the reader to Kitagawa (1964, pp. 309–311).

11 We replicated the standardization procedures by substituting the white population as the standard with the Hispanic and black populations (Appendix 1). Comparing whites against the black or Hispanic standard yields the same results presented above—the white incarceration rate would be roughly 19.5 and 12.8 % higher if whites had age-distributions equal to the Hispanic and Black populations, respectively. Perhaps more interestingly, the results suggest that the Black incarceration rate would be 11 % higher if the Black age-distribution resembled the Hispanic population (blacks would be incarcerated at an even higher rate if they were, on average, as young as the Hispanic population) and the Hispanic incarceration rate would shrink by the same magnitude if the Hispanic population resembled the black population (the results of these analyses are available upon request).
